# Associations among systemic blood pressure, microalbuminuria and albuminuria in dogs affected with pituitary- and adrenal-dependent hyperadrenocorticism

**DOI:** 10.1186/1751-0147-52-61

**Published:** 2010-11-12

**Authors:** Yu-Hsin Lien, Tsai-Yuan Hsiang, Hui-Pi Huang

**Affiliations:** 1 Azu Clinic for Animals, No. 92, Section 1, Kin-Shan South Road, Taipei 100, Taiwan; 2 Institute of Clinical Veterinary Science, National Taiwan University, No. 1, Section 4, Roosevelt Road, Taipei 100, Taiwan

## Abstract

**Background:**

Hypertension and proteinuria are medical complications associated with the multisystemic effects of long-term hypercortisolism in dogs with hyperadrenocorticism (HAC).

**Methods:**

This study investigated the relationships among adrenocorticotropic hormone (ACTH)-stimulation test results, systemic blood pressure, and microalbuminuria in clinically-healthy dogs (n = 100), in dogs affected with naturally occurring pituitary-dependent (PDH; n = 40), or adrenal-dependent hyperadrenocorticism (ADH; n = 30).

**Results:**

Mean systemic blood pressure was similar between clinically healthy dogs and dogs with HAC (*p *= 0.803). However the incidence of hypertension was highest in dogs with ADH (*p = 0.017*), followed by dogs with PDH, with the lowest levels in clinically healthy dogs (*p = 0.019*). Presence of microalbuminuria and albuminuria in clinically healthy dogs and dogs affected with HAC was significantly different (*p *< 0.001); incidences of albuminuria followed the same pattern of hypertension; highest incidence in dogs with ADH, and lowest level in clinically healthy dogs; but microalbuminuria showed a different pattern: clinically healthy dogs had highest incidences and dogs with ADH had lowest incidence. The presence of albuminuria was not associated with blood pressure values, regardless of whether dogs were clinically healthy or affected with ADH or PDH (*p *= 0.306).

**Conclusions:**

Higher incidence of hypertension and albuminuria, not microalbuminuria was seen in dogs affected with HAC compared to clinically healthy dogs; incidence of hypertension and albuminuria was significantly higher in dogs affected with ADH compared to PDH. However, presence of albuminuria was not correlated with systemic blood pressure.

## Background

Hyperadrenocorticism (HAC, Cushing’s syndrome) is a common endocrine disorder in dogs and is characterized by chronically elevated circulating concentrations of the steroid hormones produced by the adrenal cortex (e.g. cortisol) [1]. The multi-systemic effects of long-term hypercortisolism in dogs results in a variety of medical complications such as hypertension and proteinuria [1-4]. Hypertension develops by several mechanisms, including excessive renin concentrations which lead to activation of the renin-angiotensin system, increased vascular sensitivity to catecholamine, and increased concentrations of aldosterone [5-11]. These conditions warrant both prompt, in-depth evaluation of organ damage and treatment. For example, glomerular damage secondary to hypertension may further result in albuminuria which is associated with deteriorated renal function [12-17].

Apart from hypertension, many complications associated with Cushing's syndrome in humans are also linked with increased urinary albumin excretion rate. These include dyslepidaemia, reduced insulin sensitivity, and hyper-coagulative status. These complications are also associated with canine HAC [1,16,18,19].

Microalbuminuria is defined as the presence of a small amount of albumin (1 to 30 mg/dL) in the urine [16]. Microalbuminuria assays are more sensitive to albumin loss in the urine than the common urine dipstick, and urinary protein:creatinine ratio tests can detect protein loss through the urine with lower limits-of-detection than urine dipsticks. Microalbuminuria is used as an indicator in screening for early-stage renal and cardiovascular diseases in humans [12,14,17,20,21]. It is a well-known risk factor associated with deteriorated cardiovascular mortality and diabetic nephropathy in human beings [12-14,17]. Similarly, microalbuminuria is associated with renal and systemic sub-clinical diseases in dogs and cats [22-24].

The purposes of this study were to report the relationship between ACTH-stimulation tests, systemic blood pressure, microalbuminuria and albuminuria in dogs affected with naturally occurring pituitary-dependent (PDH) and adrenal-dependent hyperadrenocorticism (ADH).

## Methods

### Animals

Informed consent was obtained from all dog owners prior to the study, and number of animals of each breed in each group are shown in Table 1.

**Table 1:** Breeds and numbers of the 100 clinical healthy dogs, 40 dogs affected with pituitary-dependent hyperadrenocorticism (PDH) and 30 dogs affected with adrenal-dependent hyperadrenocorticism (ADH)

Breed	Clinically Healthy	PDH	ADH
Akita inu	0	0	1
Beagle	1	2	1
Bichon frise	0	1	0
Chihuahua	0	1	0
Cocker Spaniel	2	1	1
Japanese spitz	0	2	1
Golden retriever	2	0	0
Lhasa apso	1	0	0
Maltese terrier	20	7	9
Miniature pinscher	3	1	1
Miniature poodle	10	3	1
Miniature schnauzer	5	2	2
Mixed breed	22	7	2
Papillon	1	0	0
Pekingese	1	0	0
Pomeranian	9	3	3
Pug	1	0	0
Shetland sheep dog	1	0	0
Shihba inu	0	1	0
Shih Tzu	5	4	5
Short-haired dachshund	0	0	1
West Highland white terrier	0	1	0
Yorkshire Terrier	16	4	2

Total	100	40	30

#### Clinically healthy dogs

One hundred clinically healthy client-owned dogs visiting the National Taiwan University Veterinary Hospital from 2005 to 2009 for routine health checkups and/or annual vaccination were included in this study. Of the clinically healthy dogs, 29 were intact females, 19 were spayed females, 45 were intact males, and 7 were castrated males. The average age was 5.1 ± 3.5 years (range 0.9 to 15 years), and average body weight was 11.6 ± 9.1 kg (range 1.8 to 35.0 kg). Thorough physical examination, blood pressure measurements and routine blood work (complete blood counts and biochemical profiles) were performed to exclude heart and renal disorders that might affect blood pressure or cause microalbuminuria or albuminuria.

#### Dogs affected with hyperadrenocorticism

Medical records of 278 dogs in the National Taiwan University Veterinary Hospital during 2005-2009 with clinical signs of HAC and results of ACTH stimulation tests consistent with hyperadrenocorticism were screened. Only 70 of these records included conclusive results from an ACTH stimulation test, ultrasonographic evidence of adrenal glands, blood pressure measurements and microalbuminuria analysis. Therefore, only 70 dogs were included in the study.

Inclusion criteria of HAC were clinical signs consistent with a diagnosis of HAC (e.g., polydipsia, polyuria, polyphagia, decreased activity, panting, a potbellied appearance, and dermatologic problems), results of routine serum biochemical analyses that were consistent with a diagnosis of HAC (i.e., elevated activities of hepatic enzymes), and affirmative results of adrenocorticotropic hormone (ACTH) stimulation tests (post-ACTH cortisol concentration >20 μg/dL) [25]. Dogs with inconclusive results of ACTH stimulation tests were excluded from the study. Further differentiation between PDH or ADH was based upon adrenal ultrasonographic findings [1,26,27]. Size, shape, presence of nodular appearance and hyperechoic foci: normal or mild enlarged adrenal glands were classified as PDH; whereas adrenal glands with a nodular (or mass) appearance and hyperechoic foci were classified as ADH [1,26,27].

All included dogs were required to have had an ACTH stimulation test with intramuscular injection of 0.25 mg synthetic ACTH (Cortrosyn, Organon, The Netherlands). Informed consent was obtained from all owners prior to diagnostic testing. Blood samples for serum cortisol measurements were collected via cephalic venipuncture immediately before, and 1 hour after synthetic ACTH was injected. Serum cortisol concentrations were measured by a validated radioimmunoassay (Coat-A-Count Cortisol, Diagnostic Products Corp., U.S.A.) [27].

In this study, 70 dogs were affected with HAC, with the mean age of 11.7 ± 2.7 years and ranged from 4.5 to 18 years. Dogs had a mean body weight of 9.0 ± 7.6 kg ranging from 2.1 to 43.4 kg. Twenty-seven were spayed females, 21 were intact males, and 11 each of castrated males and intact females. Among these 70 cases, 40 dogs affected with PDH and 30 dogs affected with ADH (functional adrenal tumor). The mean age of the 40 dogs affected with PDH was 10.7 ± 2.7 years, ranging from 4.5 to 16 years with a mean body weight of 9.9 ± 8.6 kg, ranging from 2.1 to 43.4 kg. Sixteen were spayed females, 6 were intact females, 12 were intact males and 6 were castrated males. The mean age of the dogs affected with ADH was 12.9 ± 2.2 years, ranging from 8 to 18 years. This group had a mean body weight of 7.9 ± 5.6 kg, ranging from 3.0 to 24.5 kg. Eleven were spayed females, 5 were intact females, 9 were intact males, and 5 were castrated males. Breeds of 70 dogs affected with HAC are shown in Table 2.

**Table 2:** Systemic blood pressure (SBP) and status of urine albumin excretion in clinically healthy dogs and dogs affected with pituitary-dependent hyperadrenocorticism (PDH) or adrenal-dependent hyperadrenocorticism (ADH)

	Clinically healthy dogs (n = 100)	PDH (n = 40)	ADH (n = 30)
***Mean SBP (mmHg)***	152.8 ± 27.3	142.2 ± 24.9	164.1 ± 36.7†

Hypotension % (n)	1% (1/100)	0	0

Normotension % (n)	87% (87/100)	80% (32/40)	53.3% (16/30)

Hypertension % (n)	12% (12/100)	20% (8/40)	46.7% (14/30)*†

***Status of urine albumin***			

No microalbuminuria % (n)	53% (53/100)	15% (6/40)	10% (3/30)

Microalbuminuria % (n)	45% (45/100)	52.5% (21/40) *	20% (6/30) *†

Albuminuria % (n)	2% (2/100)	32.5% (13/40) *	70% (21/30) *†

* *p *< 0.05 relative to clinically healthy dogs. † *p *< 0.05 relative to PDH

### Systemic blood pressure measurement

Systemic blood pressure values of 100 clinically healthy dogs and 70 dogs affected with (pre-treated) HAC were evaluated by the same protocol--Doppler sphygmomanometry. A Doppler Ultrasonic Flow Detector (Model 811-B, Parks Medical Electronics Inc., U.S.A.) with an inflatable cuff width of 1.9, 2.5, 3, 4, or 5 cm (depending on the circumference of the antebrachium) was used. The cuff was wrapped around the middle part of the antebrachium, and a Doppler probe coated with ultrasonic transmission gel was positioned over the palmar area to detect blood flow from the arteria digitalis palmaris communis. The cuff was then inflated and deflated to obtain a systemic blood pressure reading via an aneroid pressure gauge. During systemic blood pressure measurement, the antebrachium was maintained at the level of the heart. A series of 5 readings (with 10 to 20 seconds between consecutive measurements) was obtained for each dog, and all measurements were completed within 6 minutes. To minimize procedural stress, all dogs were allowed to assume a comfortable position with only gentle restraint by their owners. The dogs remained in the same position throughout systemic blood pressure measurement. The final systemic blood pressure value was calculated as the mean of 5 readings [28]. The heart rate was manually recorded by pulse Doppler ultrasound detection over a period of 20 seconds after the systemic blood pressure value was measured. For this study, hypotension, normotension and hypertension were defined as <100 mmHg, 100-160 mmHg, and >160 mmHg, respectively.

### Microalbuminuria assay and urinary albumin:creatinine ratio

Freshly voided urine samples from 100 clinically healthy dogs and 70 dogs affected with (pre-treated) HAC were collected. Urine albumin analysis was carried out within four hours after the freshly voided urine samples were collected. Urine samples with hematuria (≥10 RBC/high power of field), pyuria (≥5 WBC/hpf), or bacteriuria was excluded after a light-microscopic examination of urine sediment. The urine albumin and creatinine concentrations were semi-quantitated (Clinitek Microalbumin Reagent Strips and Clinitek Status Analyser, Bayer Diagnostic Mfg. Ltd., UK) and urinary albumin:creatinine ratio (UACR) was calculated accordingly. In this study, no microalbuminuria, microalbuminuria and albuminuria were defined as UACR < 30 mg/g (0.03), 30-300 mg/g (0.03-0.3), and >300 mg/g (0.3), respectively.

### Statistical analysis

Comparison of systemic blood pressure between clinically healthy dogs and dogs affected with either PDH or ADH were evaluated by means of ANOVA. Relationship between post-ACTH cortisol concentrations and systemic blood pressure was analyzed by use of linear regression. Association between systemic blood pressure, microalbuminuria status (no microalbuminuria, microalbuminuria and albuminuria) and health status (clinically healthy versus HAC) were evaluated by Pearson's chi-square test. All statistical analyses were performed using commercially available software (SPSS, version 13.0, SSPS Inc, U.S.A.). Continuous data are presented as mean ± SD. Statistical significance was determined by a *p *value < 0.05.

## Results

### Systemic blood pressure and presence of microalbuminuria/albuminuria in clinically normal dogs (n = 100)

The mean systemic pressure of 100 clinically healthy dogs was 152.8 ± 27.3 mmHg. One (1%, 1/100) was hypotensive, 87% (87/100) were normotensive, and 12% (12/100) were hypertensive. Among the 100 clinically healthy dogs, 53% (53/100) did not have microalbuminuria, 45% (45/100) had microalbuminuria, and 2% (2/100) had albuminuria (Table 2).

### Systemic blood pressure and microalbuminuria/albuminuria in dogs affected with HAC (n = 70)

The mean systemic arterial pressure in dogs affected with HAC was 151.7 ± 32.1 mmHg. None was hypotensive, 68.6% (48/70) were normotensive, and 31.4% (22/70) were hypertensive. In 70 dogs with HAC, 12.9% (9/70) did not have microalbuminuria, 38.6% (27/70) had microalbuminuria, and 48.6% (34/70) had albuminuria (Table 2).

### Systemic blood pressure, presence of microalbuminuria/albuminuria and results of ACTH stimulation tests in dogs affected with PDH (n = 40)

The mean systemic blood pressure in dogs affected with PDH was 142.2 ± 24.9 mmHg. None was categorized as hypotensive, 80% (32/40) were normotensive, and 20% (8/40) were hypertensive. Among the 40 dogs affected with PDH, 15% (6/40) did not have albuminuria, 52.5% (21/40) had microalbuminuria, and 32.5% (13/40) had albuminuria. Pre- and post-ACTH cortisol concentrations in the dogs affected with PDH were 4.6 ± 2.9 (ranging from 1.9 to 14.1) and 25.5 ± 11.6 (ranging from 21.6-63.1) μg/dL, respectively (Table 2).

### Systemic blood pressure, presence of microalbuminuria/albuminuria and results of ACTH stimulation tests in dogs affected with ADH (n = 30)

The mean systemic blood pressure in dogs affected with ADH was 164.1 ± 36.7 mmHg. None was hypotensive, 53.3% (16/30) were normotensive, and 46.7% (14/30) were hypertensive. Among the 30 dogs with ADH, 10% (3/30) did not have microalbuminuria, 20% (6/30) had microalbuminuria, and 70% had albuminuria. Pre- and post-ACTH cortisol concentrations in dogs affected with ADH were 8.5 ± 6.0 (ranging from 2.9 to 28.7) and 33.2 ± 23.7 (ranging from 23.6-110.2) μg/dL, respectively (Table 2).

### Comparison of systemic blood pressure and presence of microalbuminuria/albuminuria between clinically healthy dogs and dogs affected with HAC

The mean systemic blood pressure was not different between clinically healthy dogs and dogs with HAC (*p *= 0.803). However, incidence of hypertension between clinically healthy dogs and dogs affected with HAC was significantly different (*p *= 0.019). Presence of microalbuminuria and albuminuria in clinically healthy dogs and dogs affected with HAC was significantly different (*p *< 0.001); significantly higher incidence of microalbuminuria, but significantly lower incidence of albuminuria in clinically healthy dogs compared to dogs affected with HAC. Overall, presence of microalbuminuria or albuminuria was not associated with the status of blood pressure in either clinically healthy dogs or dogs with HAC (*p *= 0.141).

### Comparison of systemic blood pressure, presences of microalbuminuria/albuminuria and results of ACTH stimulation test between dogs affected with PDH and ADH

The mean systemic blood pressure was significantly different between dogs affected with PDH and ADH (*p *= 0.004). Incidence of hypertension in dogs affected with PDH and ADH was significantly different (*p *= 0.017). Presence of microalbuminuria and albuminuria in dogs affected with PDH and ADH was significantly different (*p *= 0.007). However, albuminuria was not associated with the status of blood pressure (*p *= 0.306).

Both pre- and post-ACTH cortisol concentrations between dogs affected with PDH and ADH were significantly different (*p *< 0.001). No correlation between systemic blood pressure in either pre-ACTH (*r *= 0.095, *p *= 0.479) or post-ACTH cortisol concentrations (*r *= 0.130, *p *= 0.326) was found.

Overall, the incidence of hypertension was highest in dogs with ADH, followed by dogs with PDH, with the lowest levels in clinically healthy dogs (*p = 0.019*); incidences of albuminuria followed the same pattern: highest incidence in dogs with ADH, and lowest level in clinically healthy dogs (*p *< 0.001); but microalbuminuria showed a different pattern: clinically healthy dogs had highest incidence and dogs with ADH had lowest incidence.

## Discussion

The incidence of hypertension in dogs affected with HAC has been reported to range from 52.6 (n = 10/16) to 86.1% (n = 31/36) [3,4]. In this study, the incidence of hypertension in dogs with HAC was 46.7%, which was significantly higher than the 12% incidence observed in clinically healthy dogs, supporting previous observations that dogs affected by HAC tend to have hypertension [3,4]. However, the incidence of hypertension in dogs affected with HAC was lower than that reported in previous studies. Different methods of blood pressure measurements, definition of hypertension, and case numbers may explain lower incidence in this study. The degree of hypertension is also positively correlated with the duration of the condition rather than with cortisol concentrations [11,18]. In this study, the condition of hyperadrenocorticism was newly diagnosed. Systemic blood pressure was measured at the initial diagnosis. A higher incidence of hypertension might be seen at later stages of the condition.

In this study, higher systemic blood pressure and higher incidence of hypertension was associated with ADH compared to PDH. This finding has not been reported in dogs affected with hyperadrenocorticism. However, no correlation between the cortisol concentrations and systemic blood pressure was found. Similar results were also reported in human patients with Cushing's syndrome [11,18]. The concentration of cortisol alone may not cause hypertension. Many factors have been found to be involved in development of hypertension in human patients with Cushing's syndrome, including the duration of hypercortisolaemia, impaired microvascular reactivity, endothelial dysfunctions, and hyperaldosteronemia- associated arterial wall stiffness and fibrosis [11,18,19,29]. Higher plasma aldosterone concentrations were reported in dogs affected with ADH than in dogs with PDH [6,30]. Nevertheless, aldosterone was not involved in the development of hypertension in dogs with PDH [30]. Further study regarding association between vascular stiffness, myocardial reactivity and systemic blood pressure in dogs affected with either PDH or ADH is warranted.

Incidence of microalbuminuria in clinically healthy dogs in this study (45%) was similar to previous studies, ranging from 19 to 52% [31,32]. Surprisingly, a significantly lower incidence of microalbuminuria, but a higher incidence of albuminuria was observed in dogs affected with HAC compared to clinically healthy dogs. Microalbuminuria is a well-known risk factor associated with deteriorated renal and cardiovascular diseases in humans [12,14,17,20,21]. It is also associated with survival time in chronic renal failure [22]. Nonetheless, microalbuminuria was also found to be associated with various underlying systemic diseases, such as neoplasia, infectious diseases, and immune or inflammatory diseases [24,32]. Due to the high incidence of microalbuminuria in clinically healthy dogs and the association of this condition with various systemic disorders, the microalbuminuria test may be more useful to screen for subclinical disorders than to detect specific diseases.

In this study, incidence of hypertension and albuminuria was significantly higher in dogs affected with ADH compared to PDH. This finding has not been reported in dogs affected with HAC. The correlation between systemic blood pressure and albuminuria would be anticipated in dogs with HAC, however, this was not supported in this study. Hypertension is not the only factor associated with presence of albuminuria. Increased urinary albumin excretion rate is also linked to dyslepidaemia, reduced insulin sensitivity, impaired endothelial function and hyper-coagulative status [7]. These complications are commonly seen in both human and canine patients with Cushing's syndrome [1,16,18,19]. Glomerular changes and subsequently, increases in urinary protein excretion were found with chronic glucocorticoid use [33,34]. These may explain the disassociation between albuminuria and blood pressure in these dogs.

The cause of albuminuria in dogs affected with HAC can not be answered in this study. Nevertheless, long-term monitoring of albuminuria in terms of treatment and survival time is needed to clarify the importance of albuminuria and HAC in dogs.

## Conclusions

Incidences of hypertension and albuminuria, but not microalbuminuria, were highest in dogs affected with ADH, followed by dogs with PDH, with the lowest levels observed in clinically healthy dogs. However, the presence of albuminuria was not correlated with systemic blood pressure.

## Competing interests

The authors declare that they have no competing interests.

## Authors' contributions

YHL participated in the designs of the study and carried out the recruitment of cases of hyperadrenocorticism. She also drafted the manuscript. TYH performed measurement of systemic blood pressure, carried out the analysis of urinary albumin and creatinine, and the recruitment of clinically healthy dogs as control in this study. HPH participated in the designs of the study, carried out the recruitment of cases of hyperadrenocorticism and performed the statistical analysis and the manuscript writing. All authors read and approved the final manuscript.
